# Coexisting Remnants of the Omphalomesenteric Duct and Urachus in an Infant

**DOI:** 10.70352/scrj.cr.25-0003

**Published:** 2025-06-21

**Authors:** Noboru Oyachi, Fuminori Numano

**Affiliations:** Department of Pediatric Surgery, Yamanashi Prefectural Central Hospital, Kofu, Yamanashi, Japan

**Keywords:** omphalomesenteric duct remnant, urachal remnant, umbilical nodule, umbilical granuloma, ultrasound

## Abstract

**INTRODUCTION:**

Congenital anomalies of the umbilicus, including remnants of the omphalomesenteric duct and urachus, result from the incomplete regression of fetal structures around the 10th week of gestation. The coexistence of these anomalies in a single patient is exceptionally uncommon. This report presents the case of a neonate with an umbilical nodule and periumbilical cyst, subsequently identified as coexisting remnants of the omphalomesenteric duct and urachus.

**CASE PRESENTATION:**

This study reports the case of a 17-day-old female infant who presented with a small moist umbilical nodule and a persistent yellowish mucinous discharge. Initial treatment for umbilical granuloma failed to resolve the lesion. Imaging revealed a 2-cm cyst beneath the umbilicus and a cord-like structure connecting it to the bladder. Surgical exploration identified a 6-cm fibrous band extending from the cyst to the ileal wall, consistent with an omphalomesenteric duct remnant, and a 5-mm diameter urachal remnant connecting the cyst to the bladder. Histological analysis confirmed the presence of intestinal mucosa and transitional epithelium. The postoperative recovery of the patient was without complications.

**CONCLUSIONS:**

This case elucidates the diagnostic challenges posed by persistent umbilical lesions and highlights the importance of detailed imaging and surgical exploration for identifying rare congenital anomalies. Histopathological confirmation is essential for an accurate diagnosis. Further research is required to clarify the embryological basis and clinical implications of these anomalies.

## INTRODUCTION

Neonatal umbilical disorders commonly include umbilical hernias, nodules, and discharges. A frequent presentation is moist nodules with mild discharge, often classified as umbilical granulomas.^1)^

Although these disorders are prevalent in clinical practice, umbilical pathology includes rare but clinically significant anomalies. Among these, anomalies originate from the omphalomesenteric duct (also known as the Vitelline Duct) and urachal remnants. These anomalies occur infrequently and may present with diverse abnormalities, making diagnosis based on external appearance particularly challenging.

A notably rare presentation is the coexistence of both the anomalies in a single patient. We report a neonatal case of these dual anomalies characterized by an umbilical nodule and a periumbilical cyst.

## CASE PRESENTATION

A 17-day-old female infant was referred to our pediatric surgery department with a small, moist umbilical nodule and a yellowish mucinous discharge from the umbilicus. She was born at 38 weeks’ gestation, weighing 2714 g, with an Apgar score of 9/10. The antenatal course was uneventful. However, umbilical cord swelling of the umbilical ring was observed at birth.

On day 8, the patient underwent MRI at the hospital where she was born, which revealed no specific lesions around the umbilicus or abdominal cavity. Following umbilical cord separation, a nodule appeared at the umbilicus, which was suggestive of an umbilical granuloma. However, as the lesion did not resolve, the patient was referred to our institution for further evaluation on day 17.

**Fig. 1:**
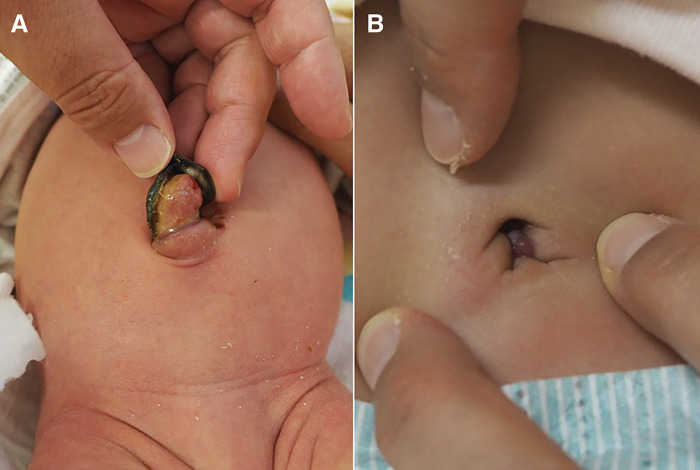
Appearance of the umbilicus on day 17 (**A**) and day 45 (**B**). (**A**) The nodule measured 1 cm in diameter and showed features consistent with umbilical granuloma. (**B**) Steroid ointment was applied as part of conservative management, resulting in partial improvement over time.

Clinical examination revealed a 1-cm umbilical nodule consistent with an umbilical granuloma (**Fig. 1A**). Following steroid ointment therapy, the lesion reduced in size, but the discharge persisted (**Fig. 1B**).

**Fig. 2:**
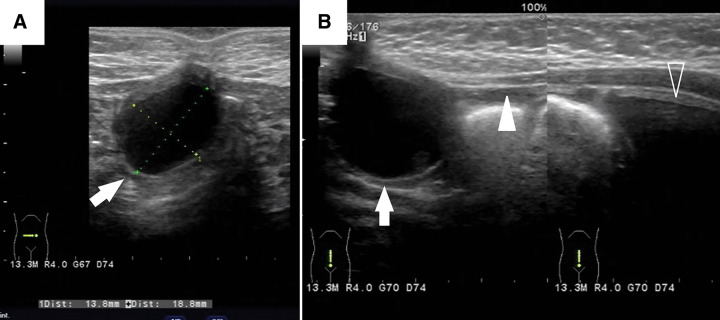
Ultrasound findings at 4 months of age. (**A**) A well-defined cyst measuring 2 cm in diameter is identified beneath the umbilicus (arrow). (**B**) A linear echogenic structure (solid arrowhead) extending from the cyst (arrow) to the urinary bladder (open arrowhead) is noted on sagittal scanning, suggesting a persistent urachal remnant.

Although the initial ultrasound results were unremarkable, a 2-cm cyst and an associated cord-like structure connecting to the bladder were identified at 4 months (**Fig. 2**). Cystourethrography demonstrated no communication between the bladder and the cyst; however, a provisional diagnosis of a urachal cyst with a patent urachus was established.

Based on these findings, elective surgical exploration through the umbilical route was considered appropriate and was performed when the patient was 4 months old.

**Fig. 3:**
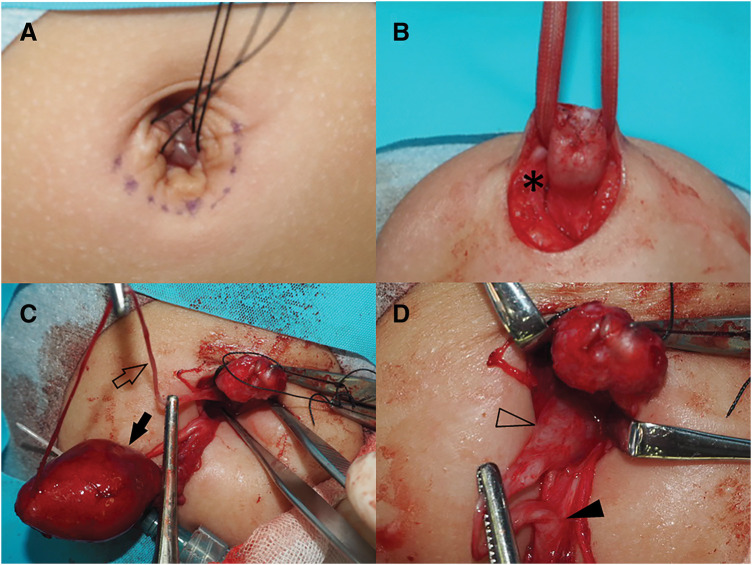
Surgical findings of coexisting remnants of the omphalomesenteric duct and urachus. (**A**, **B**) A portion of the excised umbilical nodule (asterisk) is located at the umbilicus. A curvilinear infraumbilical incision was made to access the affected area. (**C**) A 2-cm cyst (solid arrow) identified ventral to the peritoneum, appearing as a single en bloc structure with an umbilical nodule. A fibrous band (open arrow) measuring 6 cm, extending from the cyst to the antimesenteric side of the ileum, suggesting a persistent omphalomesenteric duct. (**D**) The urachal remnant (solid arrowhead), measuring 5 mm in diameter, extending from the umbilical nodule to the urinary bladder (open arrowhead).

A curvilinear infraumbilical incision was made (**Fig. 3A**, **3B**). Upon exploration, the entire nodule and a 2-cm cyst were identified, appearing as a single en bloc structure with no communication between them. The cyst was located ventral to the peritoneum, with no communication to adjacent structures. Examination of the peritoneal cavity through the umbilical incision revealed a narrow 6-cm fibrous band extending from the cyst to the antimesenteric side of the ileal wall. The band was ligated and excised at its attachment to the ileum (**Fig. 3C**).

The urachal remnant, measuring 5 mm in diameter, extended from the base of the umbilicus to the apex of the urinary bladder. It was ligated at the border of the bladder and completely excised. The specimen was confirmed to have no communication with the cyst (**Fig. 3D**). After excising all remnants, the umbilicus was reconstructed.

The postoperative course was uneventful, and the patient was discharged 6 days after surgery, with the umbilicus showing an optimal appearance.

**Fig. 4:**
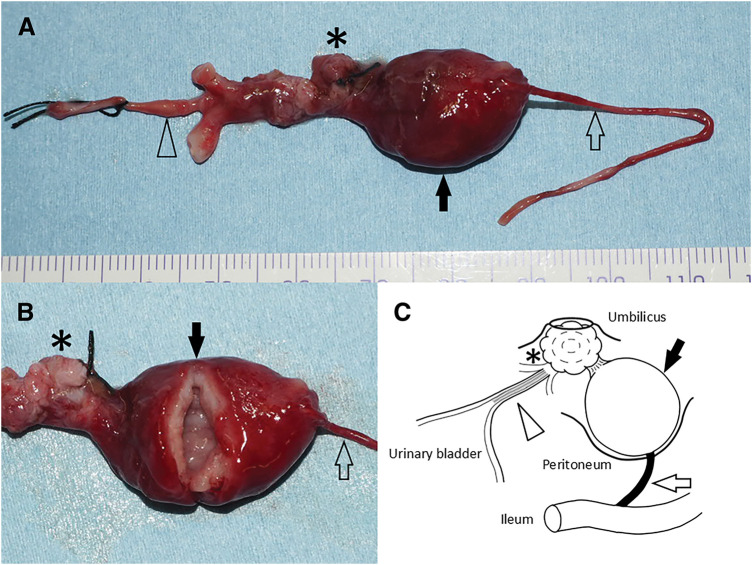
Gross findings of both remnants. (**A**) Gross appearance: The umbilical nodule (asterisk) is located at the umbilicus and associated with a cyst (solid arrow). A fibrous band (open arrow) extending from the cyst to the ileum, while the urachal remnant (solid arrowhead) is connected to the urinary bladder. (**B**) The cyst was filled with mucous fluid but lacked communication with the adjacent structures. (**C**) Schematic illustration: A diagram depicting the spatial relationships among the umbilical nodule, urachal remnant, and omphalomesenteric cyst and band.

**Fig. 5:**
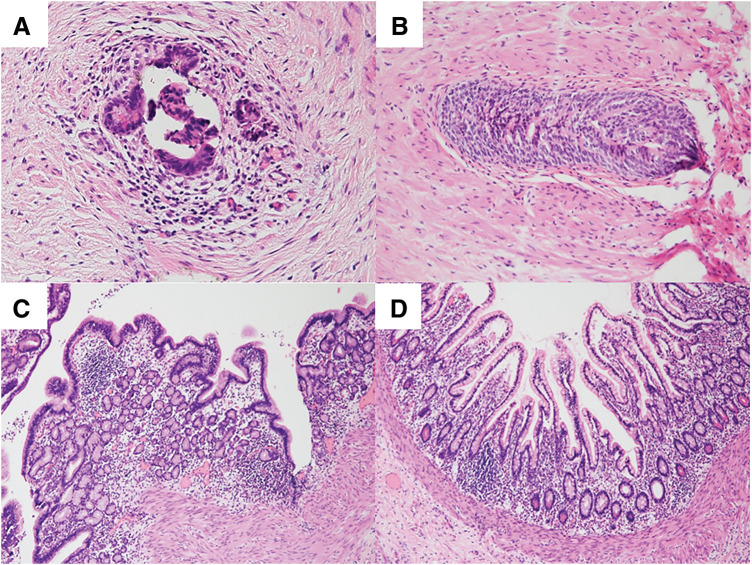
Histological findings of both remnants. (**A**) The umbilical nodule demonstrates intestinal differentiation, including Paneth cells, within the granulomatous tissue, consistent with an omphalomesenteric duct remnants. Hematoxylin and eosin (HE) staining, 200× magnification. (**B**) A tract structure extending from the nodule to the urinary bladder containing urachal epithelial tissue, indicative of a urachal remnant. HE staining, 200× magnification. (**C**, **D**) The cyst wall shows histological evidence of gastric (**C**) and small intestinal (**D**) mucosa, with the muscularis propria identified within the wall. HE staining, 100× magnification.

The gross findings for both remnants are shown in **Fig. 4**. Histological examination of the umbilical nodule revealed intestinal mucosa containing Paneth cells, consistent with an omphalomesenteric duct remnant (**Fig. 5A**). The urachal remnant, which connected the nodule to the bladder apex, was lined with the transitional epithelium and surrounded by fibrous tissue (**Fig. 5B**). The cyst contained mucous fluid and attached to the nodule exhibited both gastric and small intestinal mucosa, along with the muscularis propria, further supporting its origin in the omphalomesenteric duct remnant (**Fig. 5C**, **5D**). In addition, the band connecting the cyst to the ileum was confirmed to consist of fibrous tissue.

## DISCUSSION

Both the omphalomesenteric duct and the urachal remnants are caused by the incomplete regression of fetal structures around the 10th week of gestation. The omphalomesenteric duct remnant arises from the partial regression of the connection between the yolk sac and the midgut. This incomplete regression may result in anomalies such as Meckel’s diverticulum, fibrous bands, and umbilical lesions, including polyps, cysts, fistulas, and ectopic tissue formation.^2)^ Similarly, incomplete regression of the urachus, which connects the bladder to the umbilicus, may result in patent urachus, urachal cysts, or sinuses.^3,4)^ These anomalies typically present as umbilical discharges or masses, and often require surgical excision to prevent infection or abscess formation.

Umbilical granulomas are benign lesions commonly observed in newborns after umbilical stump separation.^1)^ It typically appears as painless, moist, pink, or red lumps at the base of the umbilicus. Granulomas result from excessive granulation tissue formation during healing, and are often accompanied by infection or fibrous tissue overgrowth.^5)^ Although they are generally harmless, untreated granulomas can secrete fluid. Treatment typically involves the application of silver nitrate to dry and shrunken tissue,^6)^ although surgical excision may be necessary in some cases.

This case highlights the rare coexistence of omphalomesenteric duct and urachal remnants, although it cannot be excluded that the simultaneous occurrence of such anomalies is underdiagnosed in general clinical practice due to missed or misinterpreted clinical findings. Initially, the patient was treated for umbilical granuloma. However, as the lesion remained unresponsive to standard therapy, further imaging evaluation prompted surgical excision, which confirmed the presence of 2 rare congenital anomalies. Initially, shortly after birth, the possibility was considered that the umbilical discharge might have been urine originating from a urachal remnant. However, as the discharge ceased shortly thereafter, its origin could not be definitively determined. In the later clinical course, the persistent discharge was presumed to have originated from the recurrent umbilical nodular lesion. The MRI performed on day 8 did not show evidence of a subumbilical cyst. We believe the cystic lesion contained minimal fluid initially, making it undetectable at that resolution. The cyst subsequently grew to approximately 2 cm over the following months, becoming apparent on ultrasound at 4 months.

**Table 1** presents 9 documented pediatric cases of coexisting remnants of the omphalomesenteric duct and urachus, along with specific umbilical findings reported in the English-language literature since 2000^7–14)^ (**Table 1**). A notable finding in our case, compared with the previously reported 8 cases, was the change in the umbilical findings over time. Specifically, the umbilical lesion initially showed signs of improvement before recurring. These anomalies likely probably arise from a shared failure of regression during fetal development, although their true incidence may be underreported.

**Table 1:** Pediatric cases of coexisting remnants of the omphalomesenteric duct and urachus, along with specific umbilical findings in English-language case reports since 2000

Author	Year	Age	Gender	Birth (GA)	Specific umbilical findings	Exams	Initial treatment attempts	Diagnosis of coexisting OMD with UR	
								OMD	UR
Kranbuhl^7)^	2007	18 months	Female	NA	Nodule/bloody purulent discharge	US	Silver nitrate application/laser treatment	Nodule	Patent urachus
Kita^8)^	2009	0 day	Male	37 weeks	Cyst/serous discharge	US	Surgical exploration	Cyst	Cyst
Sharma^9)^	2011	21 days	Female	Full-term	Prolapsed intestinal loops	NA	Surgical exploration	Patent duct	Patent urachus
Chawada^10)^	2013	6 weeks	Male	Full-term	Swelling/clear and feculent discharge	NA	Surgical exploration	Patent duct	Patent urachus
Gupta^11)^	2014	2 months	Male	Full-term	Induration/serous umbilical discharge	US/cystourethrogram	Silver nitrate application/antibiotic cream	Patent duct	Patent urachus
Bertozzi^12)^	2017	28 days	Male	Full-term	Granuloma/blood-mucinous disharge	US	Silver nitrate application	Patent duct	Patent urachus
Walia^13)^	2017	1 year	Male	Full-term	Granuloma/discharge	NA	Surgical exploration	Patent duct	Patent urachus
Ellul^14)^	2023	5 days	Female	Full-term	Lump	NA	Surgical exploration	Patent duct	Patent urachus
Present case	NA	17 days	Female	Full-term	Induration/serous discharge	US/MRI/cystourethrogram	Silver nitrate application/steroid cream	Nodule, cyst, band	Patent urachus

GA, gestational age; MRI, magnetic resonance imaging; NA, not available; OMD, omphalomesenteric duct remnant; UR, urachal remnant; US, ultrasound

Patients, ranging in age from neonates to infants, present with symptoms ranging from umbilical nodules to severe intestinal complications. Management approaches range from conservative treatments such as silver nitrate application or laser therapy^7)^ to immediate surgical exploration^8,9)^ as the first-line management approach.

When conservative methods fail, detailed diagnostic evaluation becomes essential, and ultrasonography is frequently used to confirm congenital anomalies. Surgical intervention remains the definitive approach, and most procedures are performed through umbilical incisions. Sharma et al. used a transverse subumbilical incision to resect prolapsed intestinal loops.^9)^ Histopathological analysis consistently confirmed the diagnosis and frequently identified ectopic tissues, such as pancreatic cells, within the resected nodules,^7,15)^ underscoring the complexity of these anomalies.

## CONCLUSIONS

This case elucidates the diagnostic challenges associated with persistent umbilical lesions and highlights the importance of detailed imaging and surgical exploration for identifying coexisting remnants of the omphalomesenteric duct and urachus. Timely surgical intervention remains critical to confirm the diagnosis and prevent complications of these rare congenital anomalies. Histopathological analysis is indispensable for accurate diagnosis. Additional case studies are essential to improve our understanding of embryological origins, clinical implications, diagnostic approaches, and coexistence of these anomalies.

## ACKNOWLEDGMENTS

We would like to thank Editage (www.editage.com) for English language editing.

## DECLARATIONS

### Funding

The authors declare that no funding was received for this case report.

### Authors’ contributions

NO and FN performed the operation and managed postoperative intensive care.

All authors contributed to the conception of the study and participated in its design and coordination.

NO prepared the initial manuscript draft.

All authors reviewed and approved the final manuscript.

### Availability of data and materials

The dataset supporting the conclusion of this article is included within the article.

### Ethics approval and consent to participate

Not applicable.

### Consent for publication

Informed consent was obtained from the patient's family for the publication of this case report.

### Competing interests

The authors declare that there are no competing interests regarding the publication of this paper.
